# Prevalence and associated factors of non-exclusive breastfeeding of infants during the first six months in rural area of Sorro District, Southern Ethiopia: a cross-sectional study

**DOI:** 10.1186/s13006-016-0085-6

**Published:** 2016-09-20

**Authors:** Tegegn Tadesse, Firehiwot Mesfin, Tefera Chane

**Affiliations:** 1 Gimbichu Health Center, Gimbichu, Ethiopia; 2 College of Medicine and Health Sciences, Haramaya University, Diredara, Ethiopia; 3 Wolaita Sodo University, Wolaita Sodo, Ethiopia

**Keywords:** Prevalence, Associated factors, Non-exclusive breastfeeding, Ethiopia

## Abstract

**Background:**

Despite the importance of exclusive breastfeeding, a wide number of mothers practice non-exclusive breastfeeding in Ethiopia. Therefore, this study aimed to identify prevalence and factors associated with non-exclusive breastfeeding in rural area of Sorro District in Southern Ethiopia.

**Methods:**

A community based cross-sectional study was undertaken. The study population consisted of all mothers with infants aged of 0–5 months living in the randomly selected kebeles (lowest administrative unit) in the rural area of Sorro District. The study was conducted on 602 mothers with infants selected by using systematic sampling method from 12 August to 23 August 2015. Both bivariate and multivariable logistic regression analysis were used to assess the association between the study variables and to control possible confounding.

**Results:**

The prevalence of non-exclusive breastfeeding in infants under 6 months was 49.4 %. Being currently unmarried [AOR (95 % CI) = 3.85 (1.44, 10.27)], index infant’s age being within 2–3 months [AOR (95 % CI) = 3.63 (2.06, 6.36)] and 4–5 months [AOR (95 % CI) =10.29 (5.60, 18.92)] compared to infant age 0–1 month, initiation of breastfeeding after 1 h of birth [AOR (95 % CI) = 2.11 (1.37, 3.24)], no antenatal care visit during their last pregnancy [AOR (95 % CI) =2.60 (1.64, 4.10)] and no postnatal care visit after delivery [AOR (95 % CI) = 1.90 (1.19, 3.04)] were significantly associated with non-exclusive breastfeeding.

**Conclusion:**

In this study a large proportion of mothers with infants under 6 months of age were practicing non-exclusive breastfeeding which is one of the major risks for infant and child morbidity and mortality. Taking measures on identified associated factors with non-exclusive breastfeeding was recommended to improve the status of exclusive breastfeeding in the study area.

## Background

Breastfeeding is an unequalled way of providing ideal food for the healthy growth and development of infants. Breast milk is best source of nourishment for infants and young children, and it contains antibodies that protect against some common childhood illnesses. Moreover breast milk is safe, available, affordable and one of the most effective ways to ensure child health especially in developing countries [1, 2].

The World Health Organization (WHO) and United Nation International Children’s Fund (UNICEF) recommend initiation of breastfeeding within 1 h of birth [3, 4]. According to WHO, exclusive breastfeeding is defined as the practice of feeding only breast milk (including expressed breast milk) and no other liquids or solids with the exception of drops or syrups consisting of vitamins, mineral supplements or medicine and oral rehydration solution(ORS) [3, 5, 6]. Prelacteal feeds, any fluid or food given before breastfeeding starts, are discouraged and giving colostrum is encouraged [4, 5].

Exclusive breastfeeding protects against acute and chronic diseases and promotes good growth and development. It also reduces child mortality from common childhood illnesses such as diarrhea or pneumonia and evidence shows that it protects against mother to child transmission of human immunodeficiency virus/acquired immunodeficiency syndrome (HIV/AIDS) than mixed feeding [4, 7]. If exclusive breastfeeding is universally practiced, it could save 13 % of all under five deaths [4, 8–10]. Despite its significance, less than 40 % of infants are exclusively breastfed worldwide. According to UNICEF, only 39 % of infants between 0 and 5 month old in the developing world are exclusively breastfed. About 1.45 million lives are lost due to suboptimal breastfeeding in developing countries per year [10–12]. Additionally, long term effects such as, poor academic performance, decreased productivity, and impaired cognitive and social development may be attributed to lack of exclusive breastfeeding [1, 13–15].

Ethiopia is among the nations with the highest infant and under-five mortality rates in the world and malnutrition has been attributed as a major cause of under five deaths. The government of Ethiopia has been working on improving infant and young child feeding by developing national strategies and programs based on international organizations recommendation [16]. As a result Ethiopia has noticed progress in reducing under five mortality from 2000 to 2011 by reducing malnutrition over the past decade. However, malnutrition and infectious diseases are still remaining high and highlight the need for significant investment in nutrition [10, 17, 18].

According to the Ethiopian health and demographic survey in 2011, 48 % of infants less than 6 months of age were non-exclusively breastfed even though breastfeeding is nearly universal in Ethiopia (98 %). Moreover, only 32 % or only one in three children among infants 4–5 months were exclusively breastfed. Non-exclusive breastfeeding is practiced widely and indicates Ethiopia has not yet seen the benefits of exclusive breastfeeding (EBF). Studies show that many mothers (10 to 42.9 %) start complementary feeding earlier than 6 months in Ethiopia [18–20] and prelacteal feeding is also common especially in Somali and Amhara regions (72.5 and 47.8 % respectively). Sixteen percent of infants under 6 months are bottle fed [20]. In Southern Nations and Nationality people’s region (SNNPR) the prevalence of infants under 6 months non-exclusively breastfed was not indicated according to the Ethiopia Demographic and Health Survey (EDHS) 2011 but another study revealed that 44.4 % of infants were non exclusively breastfed [16, 20].

Even though the practice of non-exclusive breastfeeding is high in Ethiopia, the information available on prevalence and factors associated with non-exclusive breastfeeding in rural communities is limited. Therefore this study aimed to identifying the prevalence and factors associated with non-exclusive breastfeeding in rural area of Sorro District, in Southern Ethiopia.

## Methods

### Study area, design and period

Sorro District is one of ten districts and one urban administration in Hadiya zone. It is located 262 km from Addis Ababa and 32 km from zonal capital, Hosanna. The total area of the District is 706 square km and lies 840–2850 m above sea level. The total population of Sorro District is 245,578 as reported by the districts finance and economic development office in 2014. There are 123,288 females accounting for 50.2 % of total population. More over the total population found in rural Sorro District is 226,190. There are 35,308 (15.6 %) under five age and reproductive age group 52,702 (23.3 %). There are 5777 (1.7 %) infants within age group 0–5 months in rural community. Estimated number of households in rural Sorro District is 11,083 households [21].

There are nine government and one non-government health centers and 46 health posts with two health extension workers at each health post. Nutrition is focused on as one of the components of health extension package.

### Source and study populations

The study was conducted using a cross-sectional design and conducted from 12 August to 23 August 2015. The study population included all mothers with infants aged 0–5 months living in rural Sorro District, and mothers with infants aged 0–5 months living in randomly selected kebeles (lowest administrative unit) were selected. Infants of age 0–5 months who were living with their biological mothers for at least 6 months in the study area were included in the study. Mothers who were severely ill and unable to respond were excluded from the study. Additionally, mothers who could not talk or hear due to disability were excluded from the study.

### Sample size determination

Sample size was determined for both objectives and the maximum sample size was determined using single proportion population formula $\left(\mathrm{n} = \frac{\left(\left(\mathrm{Z}\upalpha /2\right)2\right)\mathrm{p}\left(1\hbox{-} \mathrm{p}\right)}{\mathrm{d}2}\right)$ by considering the following assumptions: expected prevalence of bottle feeding 16 % [16], 5 % type I error, margin of error 3 and 5 % contingency for the non response and the final sample size was 602.

### Sampling techniques and procedures

Systematic sampling was carried out to undertake the study on 602 mothers with infants of age 0–5 months. First 15 kebeles were selected randomly by using a lottery method out of 46 rural kebeles found in Sorro District. Then up to date census of households which had eligible mothers with infants aged 0–5 months has been made by using health extension workers. The sampling interval was two for each Kebele. The first household was selected randomly and every second household was selected until the required number of households were achieved. When there was more than one eligible mother with an infant in the same household, one eligible mother with an infant was selected randomly by using the lottery method. When an eligible mother with an infant was not present during data collection, the household was visited for at least three times and data collected.

### Data collection method

Data were collected by using pretested structured questionnaire adapted from EDHS, Linkage project 2006 and UNICEF’s Multiple Indicator Cluster Survey Questionnaires which were designed to assess infant and young child feeding practices in developing countries. The questionnaire was translated from English to local language Hadiyigna and translated back to English by fluent speakers of the two languages. Data were collected by using face to face interviews of mother with an eligible infant based on socio demographic and economic characteristics, maternal health service related characteristics, obstetric characteristics, infant feeding practices and maternal knowledge on exclusive breastfeeding.

Data were collected by 15 diploma holder female nurses who were fluent in the local language Hadiyigna and with prior experience in data collection. Additionally four first degree health professionals supervised the data collection.

Five percent of the sample size was pretested before actual data collection period outside the selected kebeles. Intensive training was also given to data collectors and supervisors on the purpose of study, how to handle questionnaires, how to conduct data collection and on ethical consideration. Strict supervision and the overall quality of the data collection was monitored and performed by the principal investigator. The collected data were also checked for completeness and consistency before processing and analyzing data.

### Study variables and data measurement

Data were collected on non-exclusive breastfeeding considering dependent variables and on different independent variables such as, socio-demographic variables, health service related characteristics, obstetric factors (place of delivery, mode of delivery, delivery assistance and parity), feeding practice related variables (knowledge of mother on exclusive breastfeeding, early initiation of breastfeeding, giving colostrum and breastfeeding condition) and partially open ended questions were asked to know the reason why they give additional foods.

Non-exclusive breastfeeding was defined as: the proportion of infants 0–5 months of age who were not fed exclusively with breast milk in the previous 24 h with the exception of medications such as drops, syrups or ORS [22, 23].

### Data processing and analysis

The data were entered by using Epi info version 3.5.4 and exported to SPSS for windows version 21.0 to undertake the analysis. Descriptive summary was presented by using frequencies, proportions, means and tables. Household socioeconomic position was computed by using 15 variables. Household socio economic status was considered mainly based on household assets. Then principal component analysis was applied to establish wealth index which was categorized into quintile starting from lowest (poorest) to highest (richest). Both bivariate and multivariable logistic regression analysis was used to assess the association of independent variables with outcome variable and to control the possible confounding factors. Statistical tests were performed to manage missing values and to check multi-colinearity before running multivariable analysis. In order to avoid missing important variables (time of initiation of breastfeeding), the analysis was done by excluding mothers who did not remember the time of initiation. But this was done after running sensitivity analysis with three scenarios (adding mothers who didn’t remember to either group or excluding them). Hence, adding or removal of these mothers had no significant effect on the strength or the direction of association. Odds ratio with 95 % confidence interval was used to assess the strength of association. Fitness of the model was tested by checking Hosmer and Lemeshow goodness of fit test and omnibus test of model. During multivariable analysis an independent variable with p-value less than 0.05 was considered as significant and an independent predictor of non-exclusive breastfeeding.

## Result

### Socio-demographic characteristics of participants

From the total of 602 mothers with infants of age 0–5 months, 579 mothers were included in the study, making the response rate 96.2 %. The mean (±SD) age of mothers was 28.2(±4.7) years and ranges from 15 to 42 years. The median age of infants was 3 months. The majority of women, 553 (95.5 %) were protestant followers. The largest ethnic group was Hadiya 532(91.9 %) and most of the mothers 511(88.3 %) were housewives by their occupation. Ninety four percent of mothers were married during the time of survey and 334 (57.7 %) mothers were uneducated. Among all infants aged 0 to 5 months, 305 (52.7 %) infants were boys and 242 (41.8 %) were found within age group 2–3 months.

About two third of mothers 407 (70.3 %) had one up to two children of under 5 years of age and the median number of live births per mother was four which ranges from 1 up to 11 children (Table 1).

**Table 1:** Socio-demographic characteristics of mothers with infants aged 0–5months (*n* = 579), Sorro District, Hadiya Zone, Southern Ethiopia, Aug 2015

Variables		Number	Percent
Age of mother	15–20	34	5.9
	21–25	142	24.5
	26–30	259	44.7
	31–35	100	17.3
	>35	44	7.6
Religion of mother	Protestant	553	95.5
	Other^c^	26	4.5
Mother’s ethnicity	Hadiya	532	91.9
	Kambata	16	2.8
	Tambaro	30	5.2
	Other^b^	1	0.2
Mother’s educational status	No education	334	57.7
	Read and write only	83	14.3
	Pimary	135	23.3
	Secondary and above	27	4.7
Current marital status	Married	544	94.0
	Unmarried^d^	35	6.0
Occupation of mother	House wife	511	88.3
	Merchant	46	7.9
	Other^a^	22	3.8
Child’s sex	Male	305	52.7
	Female	274	47.3
Index Child’s age	0-1 m	132	22.8
	2-3 m	242	41.8
	4-5 m	205	35.4
Wealth index	Poorest	226	39.0
	Poor	67	11.6
	Middle	35	6.0
	Rich	155	26.8
	Richest	96	16.6

^a^Daily labor, craft woman, government worker ^b^Dawuro ^c^Orthodox, Catholic and Muslim, ^d^never married, divorced and separated

### Maternal health service related and obstetric characteristics

The median hour from nearby health facility (health center) was 0.75 h which ranges from 0.3 to 2.50 h. Regarding antenatal care, 363(62.7 %) mothers reported that they visited health institution for antenatal care during pregnancy of index child and 204 (56.2 %) had two to three visits. Most of the mothers 284 (78.2 %) were counseled on breastfeeding during their antenatal visits at the health institution. The majority of the mothers 481 (83.1 %) in the study area reported that they gave birth to the index child at home. During delivery, 441 (76.2 %) mothers were assisted by traditional birth attendants. Almost all mothers, 577 (99.7), had vaginal delivery and postnatal care was provided for 367 (63.4 %) mothers (Table 2).

**Table 2:** Maternal health service related and obstetric characteristics (*n* = 579), Sorro District, Hadiya Zone, Southern Ethiopia, Aug 2015

Variable		Number	Percent
Antenatal care	Yes	363	62.7
	No	216	37.3
No of antenatal visits (*n* = 363)	one	98	27.0
	two up to three	204	56.2
	four and above	58	16.0
	Don’t know	3	0.8
Counseled on breastfeeding during antenatal visits (*n* = 363)	Yes	284	78.2
	No	79	21.8
Place of delivery	Home	481	83.1
	Health facility	98	16.9
Delivery assistant	Traditional birth attendant	441	76.2
	Health professional	96	16.6
	Other^ɍ^	42	7.3
Mode of delivery	Vaginally	577	99.7
	Caesarean section	2	0.3
Received postnatal care	Yes	367	63.4
	No	212	36.6
Counseled on breastfeeding during postnatal visit (*n* = 367)	Yes	332	90.5
	No	35	9.5

The symbol "ɍ" stands for or to mean Relative, friend, neighbor and by mother herself

### Breastfeeding characteristics of participants

All mothers had ever breastfed their children at some point and they were breastfeeding their index infant during the time of the survey. About half of mothers 291 (50.3 %) initiated breastfeeding to their newborn within 1 h of delivery. One hundred eighty nine (32.6 %) mothers reported expressing and discarding the first milk (colostrum) and prelacteal feeding (anything other than breast milk given within 3 days after delivery) was given by 92 (15.9 %) respondents. Water and rue (Tena Adam in Amharic) were the commonest prelacteal feedings given to their child which accounts 39.1 and 28.3 % respectively.

Three hundred eighty four (66.3 %) mothers responded that they had breastfed the index infant eight or more times. The mean frequency of breastfeeding was 9.8 (±3.4) times.

The prevalence of non-exclusive breastfeeding among 0 to 5 months of infants was 49.4 % in the study area. This indicates additional food or fluid other than breast milk was given by 286 (49.4 %) mothers with infants of age 0–5 months. Cow’s milk and home based infant formula (“durgana”) were commonly given to these infants, reported by 53.5 and 48.3 % of respondents respectively. One hundred eighty two (63.6 %) mothers responded that a reason for giving additional food was a feeling that giving only breast milk is not enough for infant. They perceived that breast milk alone cannot satisfy their babies and does not meet the cellular requirement. They felt that breastfeeding has no adequate nutrients both in quality and quantity. According to the age specific non-exclusive breastfeeding, the prevalence of non-exclusive breastfeeding was increased from 24.2 % in 0–1 months to 69.3 % in 4–5 months. Bottle with nipple was used by 111 (19.2 %) mothers to feed index infant. The median age to cease exclusive breastfeeding was found to be 2 months in the study area.

Four hundred ninety four (85.3 %) mothers with index infant were ever heard about exclusive breastfeeding. Health extension workers were the main source of information as reported by mothers (87.2 %).

### Factors associated with non-exclusive breastfeeding

On multivariable logistic regression analysis, after adjusting for potential confounders, being currently unmarried, index infant’s age being between 2–3 and 4–5 months, not having antenatal care visit during the index pregnancy, not having post natal care visit, initiation of breastfeeding after 1 h of delivery were factors significantly associated with non-exclusive breastfeeding among mothers with infants of 0–5 months.

Those mothers who had no antenatal visits during their index pregnancy were 2.6 times more likely to practice non-exclusive breastfeeding (NEBF) than those who had antenatal care [AOR (95 % CI) = 2.60 (1.64, 4.10)]. Similarly mothers who had no postnatal care after birth of index pregnancy were 1.9 times more likely to practice NEBF than those who had postnatal care [AOR (95 % CI) = 1.90 (1.19, 3.04)]. Those mothers who had initiated breastfeeding their index infant after 1 h of delivery were nearly two times more likely to practice NEBF than those who had initiated within 1 h of delivery [AOR (95 % CI) = 2.11 (1.37, 3.24)]. The odds of NEBF were nearly four times higher among currently unmarried mothers than their counterparts [AOR (95 % CI) = 3.85 (1.44, 10.27)]. The odds of NEBF were 3.6 times higher among infants within age group of 2–3 months when compared to infants within age group of 0–1 months [AOR (95 % CI) = 3.63 (2.06, 6.36)]. Similarly the odds of NEBF were nearly ten times higher among infants within age group of 4–5 months when compared to infants age group of 0–1 months [AOR (95 % CI) =1 0.29 (5.60, 18.92)] (Table 3).

**Table 3:** Predictors of non-exclusive breastfeeding among mothers of infants of age 0–5 months (*n* = 514), Sorro District, Southern Ethiopia, Aug 2015

Variable	Non exclusive breastfeeding		COR(95 % CI)	AOR(95 % CI)
	Yes (%)	No (%)		
Antenatal care				
yes	121 (36.5)	211 (63.5)	1	1
no	123 (67.6)	59(32.4)	3.63 (2.48, 5.33)***	2.60 (1.64, 4.10)***
Postnatal care				
yes	127 (38.4)	204 (61.6)	1	1
no	117 (63.9)	66 (36.1)	2.85 (1.96, 4.14)***	1.90 (1.19, 3.04)*
Current marital status				
married	223 (46.0)	262 (54.0)	1	1
unmarried^a^	21 (72.4)	8 (27.6)	3.08 (1.34, 7.10)**	3.85 (1.44, 10.27)**
Colostrum discarded				
yes	101 (59.8)	68 (40.2)	1	1
no	143 (41.5)	202 (58.5)	0.47 (0.33, 0.69)**	0.61 (0.40, 1.03)
Time of initiation of breastfeeding				
within 1 h	109 (37.5)	182 (62.5)	1	1
after 1 h	135 (60.5)	88 (39.5)	2.56 (1.79, 3.67)***	2.11 (1.37, 3.24)***
Index infant’s age				
0–1 m	27 (22.7)	92 (77.3)	1	1
2–3 m	102 (45.5)	122 (54.5)	2.85 (1.72, 4.71)***	3.63 (2.06, 6.36)***
4–5 m	115 (67.2)	56 (32.8)	6.99 (4.09, 11.94)***	10.29 (5.60, 18.92)***
Place of delivery				
home	212 (50.7)	206 (49.3)	2.06 (1.29, 3.28)**	0.92 (0.28, 3.08)
health facility	32 (33.3)	64 (66.7)	1	1
Delivery assistance				
Health professional	30 (32.3)	63 (67.7)	1	1
Traditional birth attendant	198 (51.7)	185 (48.3)	2.25 (1.39, 3.63)***	1.58 (0.46, 5.51)
Other	22 (57.9)	16 (42.1)	1.53 (0.70, 3.32)	1.59 (0.40, 6.40)
Mother’s educational status				
no education	153 (51.9)	142 (48.1)	2.03 (0.88, 4.71)*	2.02 (0.77, 5.32)
read and write only	37 (46.8)	42 (53.2)	1.66 (0.66, 4.18)	1.53 (0.52, 4.50)
primary	45 (39.5)	69 (60.5)	1.23 (0.50, 3.00)	1.27 (0.45, 3.56)
secondary and above	9 (34.6)	17 (65.4)	1	1

Note: ***P-value < 0.001**0.001 ≤ *P* < 0.01 *0.01 ≤ *P* < 0.05 ^a^never married, divorced, separated; *COR* crude odds ratio, *AOR* adjusted odds ratio

## Discussion

The prevalence of non-exclusive breastfeeding was 49.4 % (95 % CI = 45 %, 53 %) in this study. The finding is similar with the Ethiopian national study (48 %), Jima Arjo District (52.1 %) and Egypt (47 %) [20, 24, 25]. However, the prevalence of NEBF in this study was lower than the findings in other developing countries like 88 % in Cote d’Ivoire, 80 % in Cameron, 62 % in Burkina Faso, 83.6 % in Nigeria, 68 % in Kenya and 62.9 % in Pakistan [26–28]. In contrast to this, the current study finding is higher than findings in East Ethiopia (28.3 %), South Eastern Ethiopia (28.7 %) and Southern Ethiopia (44.4 %). It is also higher than studies done in Uganda (33 %) [16, 26, 29, 30]. This might be due to methodological differences in selecting target groups and variations in the definition of exclusive breastfeeding and presence of socio cultural differences among study subjects.

According to age specific non-exclusive breastfeeding, the prevalence of NEBF was increased from 24.2 % in 0–1 months to 69.3 % in 4–5 months of age. This finding is in line with Ethiopian national study which was increased from 30 % at infant’s age of 0–1 months to 68 % at age of 4–5 months. However, the finding is higher than a study conducted in Goba, South Eastern Ethiopia which stated that NEBF was increased from 16.8 % in 0–1 months to 35.1 % in 4–5 months [20, 30]. This might be due to differences in understanding of optimal breastfeeding among study respondents. Since the study was conducted in a rural area, behavioral change is not achievable within short time by only health extension workers. Advocacy of the optimal child feeding practice through mass media communication may not successfully reach this rural community.

Multivariable logistic regression showed that age of infant was an independent predictor of NEBF in the study area. As the age of infant approaches to 6 months, the prevalence of non-exclusive breastfeeding was increased. It is in line with study conducted in Goba, South Eastern Ethiopia [30]. It is also consistent with studies done in China, Pakistan, Nigeria, Egypt and Uganda [24, 27, 28, 31, 32]. This could be explained by mothers assuming breast milk alone would not be sufficient for infants once they are older than 2 months. Greater than 60 % of the respondents perceived that breast milk is insufficient for normal child growth and development. In addition to this, it may be correlated with high levels of maternal illiteracy (about 60 %) and low postnatal care follow up significantly affecting exclusive breastfeeding in the study area.

Unmarried mothers were nearly four times more likely to breastfeed non-exclusively than those mothers who were married. This finding is consistent with another study done in Ethiopia [33]. This might be explained by the fact that unmarried mothers lack support given by their husbands and other family members on infant feeding practices.

This study also revealed that lack of early initiation of breastfeeding was associated with NEBF. The finding goes in line with the studies conducted in rural Egypt and Mecha District,North Western Ethiopia [13]. This might be explained by mothers’ tendency to commence complementary feeding early if the initiation of breastfeeding is delayed after delivery. Not having antenatal care during their last pregnancy was associated with NEBF in this study. This is similar to findings in Nigeria and Egypt [24, 28], possibly because mothers who did not receive antenatal care lacked information on optimal infant feeding, especially exclusive breastfeeding, given by health workers at health institutions. Finally this study revealed that mothers who had not received postnatal care were more likely to practice NEBF than their counterparts. This finding is in line with study finding in Mecha District, North Western Ethiopia [13]. This might also be due to the fact that mothers who did not receive postnatal care missed out on advice about exclusive breastfeeding from health workers during the contact.

This study shows that maternal and infant socio-demographic factors such as, sex of infant, educational status of mother and occupation of mother did not show significant association with NEBF unlike earlier studies [20, 24, 27, 28, 30, 34–36].

Household socio-economic status also did not show significant association with NEBF [27, 28, 33, 36]. This might be due to the fact that studies conducted in Ethiopia are not considering different variables enough to explore and assess household socio-economic status.

This study used 24-h recall method which underestimates the prevalence of NEBF. The method misclassifies many mothers as exclusively breastfeeding in which those mothers who have given liquids or other substances other than breast milk regularly may not have given them in the previous 24 h, and thus underestimates NEBF [30, 31].

Maternal recall may also affect median duration of NEBF which is prone to maternal recall bias. We were unable to assess maternal knowledge about EBF or household wealth due to insufficient data. Living conditions were not assessed in detail.

## Conclusion and recommendations

This study concludes that the prevalence of NEBF is higher according to WHO and Ethiopian National recommendations on infant and young child feeding. It indicates that greater effort should be invested on reducing the level of NEBF in Sorro District. Non-exclusive breastfeeding was common among unmarried mothers, initiated breastfeeding after 1 h of delivery, who had no antenatal or postnatal care and infants’ age was over 2 months. Women in the study area need support to have an appropriate infant feeding practice.

Based on the study findings the following recommendations were forwarded to the District health office and regional health bureau:

Every effort should be made for mothers having infants within age group 2–3 and 4–5 months to be at the track of infant and young child feeding recommendation for only feeding breast milk until the first 6 months.

Unmarried women should be provided more support to exclusively breastfeed infants until the first 6 months.

Nutrition education on early initiation of breastfeeding should be strengthened by educating mothers in both community and health institutions.

Strengthening and promoting antenatal and postnatal services by health workers and district health office is recommended to address all eligible mothers with infants.

## Abbreviations

AOR: Adjusted odds ratio
CI: Confidence interval
COR: Crude odds ratio
EBF: Exclusive breastfeeding
EDHS: Ethiopia Demographic and Health Survey
NEBF: Non-exclusive breast feeding
NORAD: Norwegian agency for development cooperation
ORS: Oral rehydration solution
SNNPR: Southern Nations Nationality People’s Region
